# Anesthesia management of CRS and HIPEC in advanced ovarian cancer with ultra-high intra-abdominal pressure: a case report

**DOI:** 10.3389/fonc.2024.1449171

**Published:** 2024-11-27

**Authors:** Suli Chen, Yanjun Lin, Shuncai Gao, Shuo Liu, Zhanmin Yang, Ruiqing Ma, Liangyuan Lu

**Affiliations:** ^1^ Department of Anesthesiology, Aerospace Center Hospital, Peking University Aerospace School of Clinical Medicine, Beijing, China; ^2^ Department of Myxomatology, Aerospace Center Hospital, Peking University Aerospace School of Clinical Medicine, Beijing, China

**Keywords:** ovarian cancer, cytoreductive surgery, hyperthermic intraperitoneal chemotherapy, intra-abdominal hypertension, anesthetic management, goal-directed fluid therapy, temperature

## Abstract

Cytoreductive surgery (CRS) combined with hyperthermic intraperitoneal chemotherapy (HIPEC) is a leading treatment for advanced ovarian cancer, significantly improving overall survival and disease-free survival. This case involves a patient with peritoneal metastasis and ultra-high intra-abdominal pressure (36 mmHg). CRS + HIPEC induces extensive pathological and physiological changes affecting respiratory, circulatory, renal, coagulation, and metabolic systems. Effective perioperative anesthesia management, including the type and volume of fluids administered, is crucial for optimizing patient outcomes. The complexities of anesthesia management in such cases present significant challenges.

## 1 Introduction

Cytoreductive surgery (CRS) combined with hyperthermic intraperitoneal chemotherapy (HIPEC) is a critical treatment modality for advanced ovarian cancer, known to significantly improve overall survival and disease-free survival rates. The procedure involves extensive surgery to remove tumor deposits, followed by the application of heated chemotherapy directly into the abdominal cavity, aiming to eradicate microscopic cancer cells and reduce recurrence.

In this report, we present a case of a 70-year-old female patient with advanced ovarian cancer and ultra-high intra-abdominal pressure, highlighting the complexities and challenges of anesthesia management during CRS and HIPEC.

## 2 Case presentation

**Figure 1:**
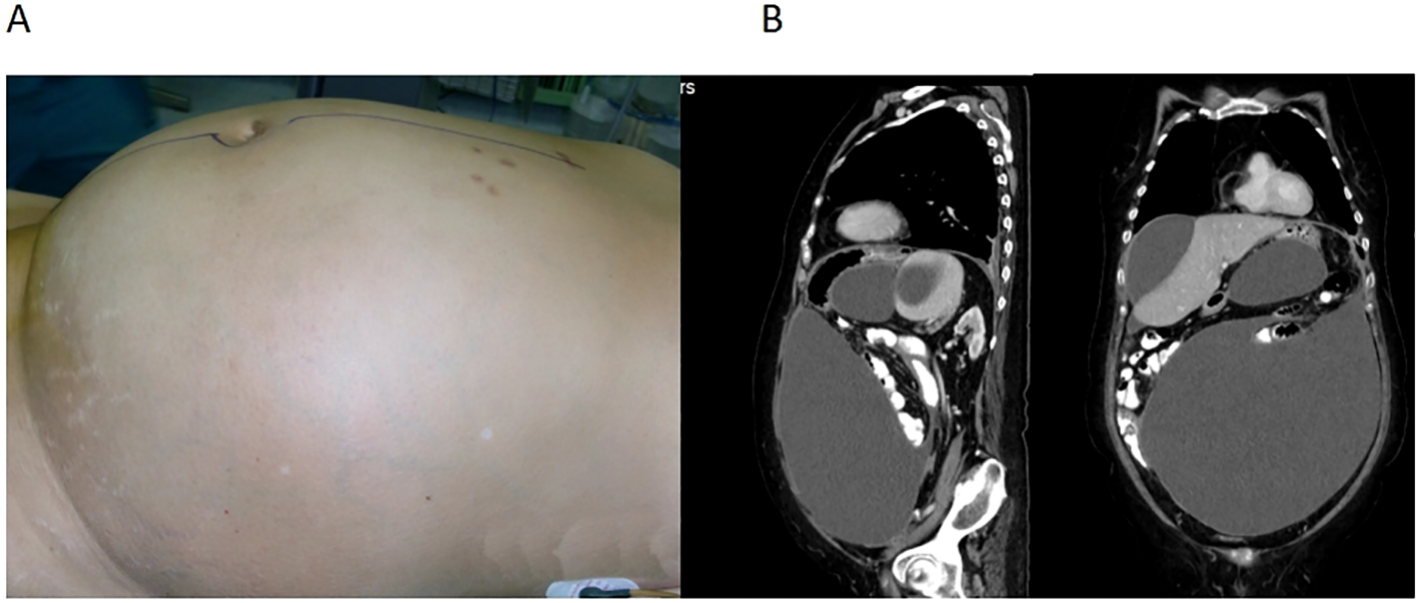
**(A)** A large abdominal circumference of 121 cm. **(B)** Computed tomography showing cystic solid nodule in the right adnexal area, diffuse thickening of the peritoneum and greater omentum, and multiple encapsulated fluid accumulation in the abdominal and pelvic cavities.

A 70-year-old woman (height, 168 cm; weight, 84 kg) was admitted with increased abdominal circumference and intermittent vomiting. The patient provided written consent to publish this case. Four months prior, deep vein thrombosis was detected in both lower limbs, and multiple pulmonary embolisms were found in both lungs 1 month before admission, necessitating the implantation of inferior vena cava filters and initiation of oral anticoagulant therapy. Her abdominal circumference was 121 cm (**Figure 1A**). Pathological analysis of the ascitic fluid revealed abdominal mucinous adenocarcinoma. The thrombus was stable, and CRS combined with HIPEC was planned.

The patient had a history of lacunar cerebral infarction and previous surgeries, including cholecystectomy and knee joint replacement. Vascular ultrasound confirmed deep vein thrombosis in both lower limbs. Pulmonary CTA revealed multiple embolisms in both pulmonary artery branches. Pelvic and abdominal CT scans showed a cystic solid nodule in the right adnexal area, diffuse thickening of the peritoneum and greater omentum, and multiple encapsulated fluid accumulations in the abdominal and pelvic cavities (**Figure 1B**). Preoperative colonoscopy indicated fixed adhesions in the left colon, likely due to external pressure from a protruding sigmoid colon. Electrocardiography revealed sinus tachycardia and an incomplete right bundle branch block. The left ventricular ejection fraction was 60%. Coagulation function: INR was 1.01, PT was 11.1 s; glomerular filtration rate was 58.50% ml/min/1.73 m^2^, albumin was 31.3 g/L, and other indicators were generally normal. Arterial blood gas analysis is presented in **Table 1**.

**Table 1:** Blood gas analysis results.

Project/Time	Preoperative	Before anesthesia	After draining ascites	After CRS	After HIPEC
PH	7.45	7.43	7.38	7.40	7.36
FiO_2_%	21	21	50	80	80
PaO_2_ mmHg	108	77	156	195	184
PaO_2/_FiO_2_	372	366	312	244	230
PaCO_2_ mmHg	42	34	39	39	38
K+ mmol/L	3.4	2.9	3.5	3.9	3.6
Ca++ mmol/L	1.14	1.17	1.17	1.19	1.09
Lac mmol/L	0.9	1.0	0.8	0.7	0.7
BE mmol/L	5.7	-1.2	-1.8	-0.5	-3.6
Hb g/dl	11.2	11.9	9.4	8.7	7.7

### 2.1 Anesthesia management

The patient was positioned semi-recumbent with the operating table adjusted to a 45° head-up and foot-low position with a 30° left tilt. Standard monitoring included electrocardiogram, direct arterial blood pressure (IBP), SpO2, oropharyngeal temperature, BIS, and FloTrac Vigileo system for CI and SVV. Initial vital signs were as follows: arterial blood pressure 120/72 mmHg, heart rate 112 beats/min, and SpO2 93%. Pre-anesthesia arterial blood gas analysis is shown in **Table 1**. Pre-induction, 100 ml of gastric juice was aspirated, and oxygen was administered at 7 L/min for 10 min. Intravenous anesthesia induction included 25 mg of propofol, 15 μg of sufentanil, and 50 mg of rocuronium bromide. A 7.0 tracheal catheter was inserted under visual laryngoscopy after 1 min. Anesthesia was maintained with a combination of intravenous and inhalational agents, with BIS maintained between 40 and 60. A right internal jugular vein catheter was inserted under ultrasound guidance to monitor central venous pressure.

### 2.2 Cytoreductive surgery

**Figure 2:**
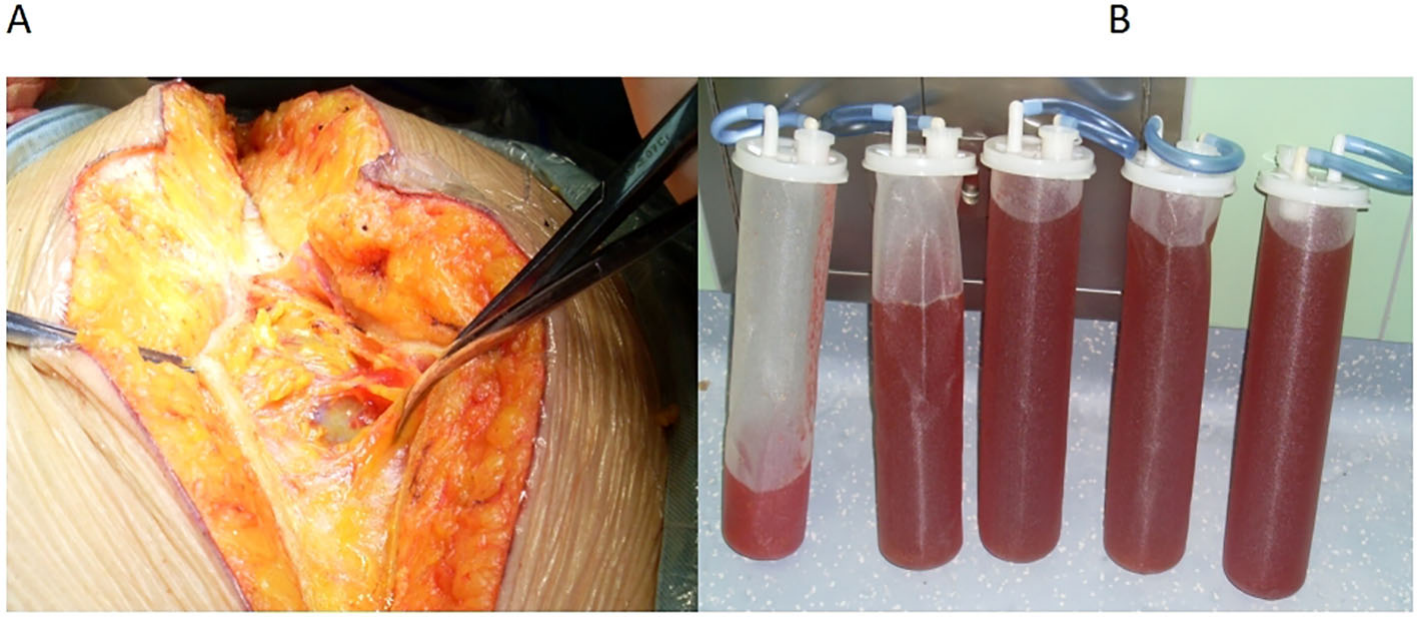
**(A)** A small incision of approximately 0.5 mm in the peritoneum. **(B)** The ascitic fluid of the patient.

**Figure 3:**
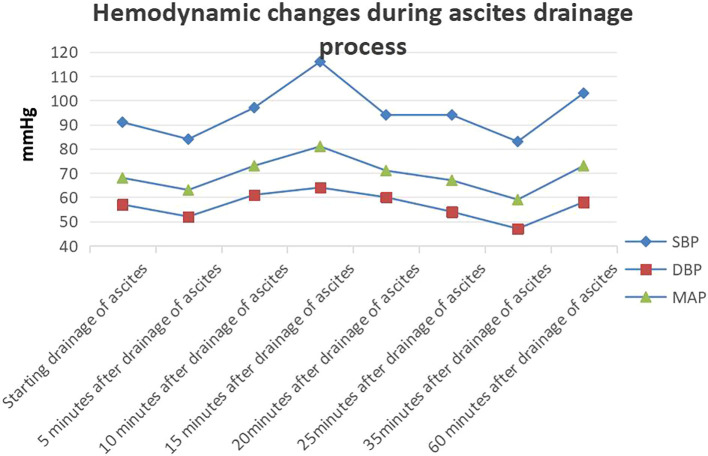
Hemodynamic changes during the ascites drainage process.

Before incision, we inserted an arterial puncture needle into the abdominal cavity at the two transverse fingers above the navel. Then connected a disposable pressure sensor (AT 4812-3, BIOPTIMAL INTERNATIONAL PTE. LTD) to measure intra-abdominal pressure. The zero point was set at the fourth intercostal space of the midaxillary line.Intra-abdominal pressure before incision was 36 mmHg. The surgeon made a small incision (approximately 0.5 mm) in the peritoneum, discharging 8000 ml of abdominal fluid within 25 min (**Figures 2A, B**). During this period, 100 ml of lactated Ringer’s solution and 500 ml of succinyl gelatin were administered. Peak airway pressure decreased from 33 cmH_2_O to 23 cmH_2_O, CI increased from 1.9 L/min/m^2^ to 2.1 L/min/m^2^, SVV decreased from 15 to 7, and CVP decreased from 23 cmH_2_O to 15 cmH_2_O. Blood pressure and heart rate remained stable (**Figure 3**). The tumor reduction process lasted approximately 5 h. Outputs during this period included 1500 ml of blood and 1200 ml of urine, while inputs included 2500 ml of lactated Ringer’s solution, 1000 ml of succinyl gelatin, and 300 ml of 20% albumin. Nasopharyngeal temperature decreased from 36.2°C to 35.7°C.

### 2.3 Hyperthermic intraperitoneal chemotherapy

Following CRS and achieving sufficient hemostasis, silicone tubes were placed in the left and right diaphragmatic, pelvic, and right iliac fossa, and the skin was sutured. The circulating hot infusion machine monitored the inlet water temperature at 44°C and the outlet water temperature at 41°C. The infusion solution comprised 4000 ml of physiological saline with 80 mg cisplatin and 80 ml of elemene. Infusion time was 60 min, during which the nasopharynx temperature rose to 37.4°C, and abdominal cavity temperature ranged from 42.4°C to 42.7°C. Urine output was 600 ml.

During surgery, low-dose norepinephrine was continuously administered to maintain hemodynamics. Blood gas analyses were conducted after ascitic drainage, CRS, and HIPEC (**Table 1**). Post-HIPEC blood gas analysis showed a Hb level of 7.6 g/dl, necessitating the administration of 2 units of packed red blood cells and 400 ml of fresh frozen plasma. Post-surgery, the patient was transferred to the ICU with a tracheal catheter, which was removed 2 h later. The patient was then transferred to the general ward the next morning. The blood gas analysis at different times during the surgery is shown in **Table 1**.

### 2.4 Pathology

The pathology report confirmed bilateral ovarian mucinous adenocarcinoma with cancer tissue invading the serosal surfaces of both fallopian tubes. Adenocarcinoma was also observed in the fibrous adipose tissue of the left peritoneum and residual omentum and in the right peritoneum and omentum.

### 2.5 Postoperative care and follow-up

The patient underwent five sessions of intraperitoneal hyperthermic perfusion chemotherapy (3500 ml of physiological saline + 1g of fluorouracil) from the second to the sixth day post-surgery. The drainage tube was removed on the 12th day. Postoperative complications included pneumonia and pleural effusion, which were managed with anti-infection therapy, closed thoracic drainage, and enhanced nutritional support. The patient was discharged on the 21st day post-surgery. However, the patient experienced a recurrence of pneumonia a week after discharge and succumbed to respiratory failure 45 days post-surgery.

## 3 Discussion

The patient presented with a high intra-abdominal pressure of 36 mmHg, posing challenges during anesthesia induction, particularly the risk of reflux aspiration. Studies have highlighted the efficacy of rapid sequential induction intubation in reducing this risk (1). Additionally, the presence of ascites and elevated intra-abdominal pressure necessitated careful evaluation and preparation for potentially difficult airway conditions. Measures such as pre-oxygenation for 10 min (7 L/min), head elevation, and a left tilt positioning were implemented to mitigate these risks.

During CRS + HIPEC, a significant amount of fluid transfer occurs, averaging approximately 10–12 ml/kg/h (2). This fluid shift results from several factors: extensive drainage of ascites, tumor reduction, peritoneal resection, blood loss, and the vasodilation and increased capillary permeability induced by the high fever during HIPEC. Recent studies have underscored the benefits of targeted fluid therapy in noncardiac surgery (3, 4). To achieve this, we employed the Flotac/Vigileo hemodynamic monitoring system to target CI and SVV. Despite the patient’s elevated intra-abdominal pressure, weakened myocardial contractility, and poor tolerance to large fluid volumes, we addressed insufficient intravascular volume by administering a low dose of norepinephrine (<0.1μg/kg/min) to maintain vascular tone and ensure adequate organ perfusion. This intervention resulted in a decrease in lactic acid levels from 1.0 mmol/L before anesthesia to 0.7 mmol/L after thermal perfusion.

The primary objective of liquid therapy during CRS + HIPEC is to sustain normal blood volume and prevent a decline in plasma osmotic pressure. Although the choice of liquids, particularly colloidal solutions, remains contentious, guidelines (5) advocate for albumin supplementation in cases of hypoalbuminemia resulting from extensive ascites and blood loss. However, further research is required to determine the optimal timing and dosage of albumin supplementation. A study by Wiedermann (6) supports the beneficial role of albumin in restrictive fluid therapy. In our patient who presented with 8000 ml of ascites, we supplemented a total of 60 g of 20% albumin during surgery. Hemodynamics remained stable throughout the procedure, and urine output reached 1800 ml. Postoperatively, the mean albumin level was 30.1 g/L.

Patients undergoing CRS + HIPEC experience drastic temperature fluctuations (7). Prolonged surgery, intravenous infusion, and tumor reduction predispose patients to hypothermia during CRS. Conversely, the infusion of a 44°C solution into the abdominal cavity during HIPEC rapidly raises body temperature. However, core temperatures exceeding 39°C can precipitate physiological complications, including organ damage (8). To manage temperature dynamics effectively, we utilized a heating blanket and heater during CRS and adjusted the room temperature to 20°C during HIPEC. Additionally, we employed an ice pack on the neck and administered 20–22°C fluids during HIPEC. Notably, the patient’s nasopharyngeal temperature peaked at 37.4°C, while abdominal temperature ranged from 42.4°C to 42.7°C. Maintaining normothermia is critical for successful perioperative management of CRS + HIPEC. Foam-based intraperitoneal chemotherapy (9) and intraperitoneal hyperthermia and dehydration (10) provide new treatment options for peritoneal cancer patients. These two are promising treatment methods in the field of peritoneal cancer, but currently our center does not have the conditions to carry them out. We will closely monitor the research progress of this research team. However, the impact of increased abdominal pressure undergoing HIPEC on circulation and metabolism cannot be ignored. We did not monitor intra-abdominal pressure undergoing HIPEC, but judging from the degree of abdominal distension, the intra-abdominal pressure during HIPEC did not exceed the preoperative intra-abdominal pressure.

Acute kidney injury and coagulation disorders (11) were significant perioperative concerns for our patient. With a preoperative intra-abdominal pressure of up to 36 mmHg, decreased renal perfusion, and a reduced glomerular filtration rate of 58.50% ml/min/1.73 m^2^, the patient was at heightened risk. However, postoperatively, blood creatinine levels decreased from 86.8 μmol/L to 64.1 μmol/L. This improvement may be attributed to the reduction in intra-abdominal pressure post-surgery, enhancing renal perfusion, and individualized intraoperative anesthesia management. Our liquid therapy strategy focused on isotonic crystal fluids, with limited gelatin usage, and included albumin infusion to maintain osmotic pressure. Perioperative albumin infusion, guided by CRS + HIPEC guidelines, did not elevate the risk of renal failure. Attention must be paid to intraoperative blood loss, degree of blood dilution, and coagulation function, especially when blood loss exceeds 50% of the blood volume, necessitating active prevention and treatment of coagulation disorders.

Despite implementing protective ventilation strategies and reducing inhaled oxygen concentration, our patient experienced postoperative pulmonary complications. These complications likely stem from factors such as prolonged surgical duration, inflammatory damage, and mechanical ventilation-induced trauma.

## 4 Conclusion

Understanding the pathological and physiological changes during CRS + HIPEC and implementing individualized anesthesia management strategies are essential for maintaining the patient’s physiological stability. Our goal-oriented liquid therapy, targeting CI and SVV and supplemented with continuous low-dose norepinephrine infusion, effectively minimized renal dysfunction due to insufficient blood volume and prevented coagulopathy from excessive fluid volume. We successfully maintained normal body temperature during surgery and closely monitored and managed coagulation function. The findings suggest that tailored fluid management and vigilant monitoring can significantly improve patient outcomes during complex procedures like CRS + HIPEC. Future studies should focus on optimizing fluid therapy protocols and further exploring the benefits of individualized anesthesia management to enhance patient safety and recovery.

## Ethical approval

This study was approved by the Institutional Research Ethic Committee of the Aerospace Center Hospital (No. 2021-QT-014).

## Data Availability

The original contributions presented in the study are included in the article/supplementary material. Further inquiries can be directed to the corresponding authors.
